# Inhibition of autophagy enhances the antitumour activity of tigecycline in multiple myeloma

**DOI:** 10.1111/jcmm.13865

**Published:** 2018-09-24

**Authors:** Ruye Ma, Yu Zhang, Wei Wang, Junqing Wu, Qianqian Yang, Wanling Xu, Songfu Jiang, Yixiang Han, Kang Yu, Shenghui Zhang

**Affiliations:** ^1^ Department of Hematology Wenzhou Key Laboratory of Hematology The First Affiliated Hospital of Wenzhou Medical University Wenzhou China; ^2^ Department of Hematology Taizhou Municipal Hospital Taizhou China; ^3^ Central Laboratory The First Affiliated Hospital of Wenzhou Medical University Wenzhou China; ^4^ Division of Clinical Research The First Affiliated Hospital of Wenzhou Medical University Wenzhou China

**Keywords:** autophagy, cell cycle, multiple myeloma, tigecycline

## Abstract

Accumulating evidence shows that tigecycline, a first‐in‐class glycylcycline, has potential antitumour properties. Here, we found that tigecycline dramatically inhibited the proliferation of multiple myeloma (MM) cell lines RPMI‐8226, NCI‐H929 and U266 in a dose and time‐dependent manner. Meanwhile, tigecycline also potently impaired the colony formation of these three cell lines. Mechanism analysis found that tigecycline led to cell cycle arrest at G0/G1 with down‐regulation of p21, CDK2 and cyclin D1, rather than induced apoptosis, in MM cells. Importantly, we found that tigecycline induced autophagy and an autophagy inhibitor bafilomycin A1 further amplified the tigecycline‐induced cytotoxicity, suggesting that autophagy plays a cytoprotective role in tigecycline‐treated MM cells. Mechanisms modulating autophagy found that tigecycline enhanced the phosphorylation of AMPK, but did not decrease the phosphorylation of Akt, to inhibit the phosphorylation of mTOR and its two downstream effectors p70S6K1 and 4E‐BP1. Tigecycline effectively inhibited tumour growth in the xenograft tumour model of RPMI‐8226 cells. Autophagy also occurred in tigecycline‐treated tumour xenograft, and autophagy inhibitor chloroquine and tigecycline had a synergistic effect against MM cells in vivo. Thus, our results suggest that tigecycline may be a promising candidate in the treatment of MM.

## 1 INTRODUCTION

Multiple myeloma (MM) is characterized by the accumulation of malignant plasma cells in the bone marrow and usually accompanied by the secretion of monoclonal immunoglobulins that are detectable in serum or urine.^1^ Combined with autologous stem cell transplantation and improvements in supportive care, the employment of novel drugs such as proteasome inhibitors, immunomodulatory agents and monoclonal antibodies has effectively improved response and substantially enhanced overall survival in the past decade.^2^, ^3^, ^4^ However, drug resistance resulting in relapse commonly occurs and MM remains an incurable disease. Therefore, novel therapies are urgently needed.

Tigecycline is the first member of a new generation of tetracyclines called glycylcyclines approved by the FDA in 2005, which is a broad spectrum antibiotic used for the treatment of bacterial infections. The mechanism of action is that tigecycline can inhibit bacterial protein synthesis by binding to the 30S ribosomal subunits.^5^ Beyond its role as an antimicrobial, accumulating evidence shows that tigecycline has anticancer properties. It can inhibit the growth and metastasis of multiple tumour cells, including acute myeloid leukaemia,^6^ gastric cancer,^7^ melanoma,^8^ neuroblastoma,^9^ cervical squamous cell carcinoma 10 and glioma.^11^ The anticancer mechanism of tigecycline appears to vary in different tumour types. Besides the inhibition of mitochondrial protein synthesis, other mechanisms including autophagy have been found to be involved in antitumour effects.^7^

Autophagy, or cellular self‐digestion, is a cellular process by which the cell ensures sufficient metabolites by breaking down its own organelles and cytosolic components when nutrients become limiting.^12^ A growing evidence demonstrates that autophagy is involved in development, differentiation and tissue remodelling in various organisms.^13^ Autophagy is also implicated in certain human diseases including inflammation, neurodegeneration and cancer.^14^ Paradoxically, autophagy can contribute to cell damage but may also serve to protect cells. When autophagy occurs, microtuble‐associated protein light chain 3‐I (LC3‐I) is converted to the membrane‐bound form (LC3‐II), which is associated with autophagic vesicles and exhibits classical punctate distribution, as classical protein markers of autophagy.^15^ Meanwhile, p62/sequestosome‐1 (SQSTM1) is degraded following an increase in autophagic flux for which this protein presently serves as another classical hallmark.^16^

Mammalian target of rapamycin (mTOR) as an evolutionarily conserved serine/threonine kinase has two structurally and functionally distinct complexes termed mTOR complex 1 (mTORC1) and mTOR complex 2 (mTORC2), which can tightly regulate autophagy.^17^ AMP‐activated protein kinase (AMPK) is one of the major stress‐sensing enzymes and can actively regulate metabolism and cell proliferation. Prominently, AMPK is also a critical regulator of autophagy. Phosphorylation of AMPK results in inhibition of mTOR, which activates autophagy.^18^

In this study, we have demonstrated that tigecycline significantly inhibits the proliferation and colony formation of MM cell lines RPMI‐8226, NCI‐H929 and U266 by inducing cell cycle arrest at G0/G1 phase. Additionally, autophagy also plays a cytoprotective role in tigecycline‐induced MM cells, and combination with chloroquine and tigecycline synergistically inhibits the tumour cell growth in a mouse xenograft model of RPMI‐8226 cells.

## 2 MATERIALS AND METHODS

### 2.1 Antibodies and reagents

Tigecycline was purchased from Sigma‐Aldrich (St.louis, MO). Bafilomycin A1 (Baf A1) was purchased from Selleck Chemical (Houston, TX). The above agents were prepared in phosphate‐buffered saline (PBS). The antibodies against LC3, SQSTM1/p62, p21, cyclin D1, CDK2, AMPKa, p‐AMPKa (Thr172), mTOR, p‐mTOR (Ser2448), p70 ribosomal S6 kinase (p70S6K), p‐p70S6K (Thr389), 4E‐binding protein 1 (4E‐BP1), p‐4E‐BP1 (Thr37/46), GAPDH were purchased from Cell Signaling Technology (Beverly, MA).

### 2.2 Cell viability assay

Human MM cell lines RPMI‐8226,NCI‐H929 and U266 were cultured in RPMI‐1640 medium supplemented with 8% fetal bovine serum in a humidified atmosphere containing 5% CO_2_ at 37°C. The cell viability was determined using the Cell Counting Kit‐8 (CCK‐8) assay according to the manufacturer's protocol (Dojindo, Kumamoto, Japan). Briefly, RPMI‐8226, NCI‐H929 or U266 cells were seeded at a density of 8 × 10^3^/well in 96‐well plates and exposed to tigecycline at different concentrations (0, 10, 20, 40 μmol/L) for 24, 48 and 72 hours. The absorbance (A) was measured at 450 nm using an ELISA reader (ELx800, Bio‐Tek Instruments, Winooski, VT, USA). The Cell viability rate (%) = A450, _tigecycline_/A450, _control_ × 100%.

### 2.3 Colony formation assay

Multiple myeloma cells were seeded at about 1 × 10^4^ cells/well in 6‐well plates with or without tigecycline (20 μmol/L) in methylcellulose medium (MethoCult™ H4034, Stemcell Technologies, Vancouver, BC, Canada). After incubation for 7 days in a 5% CO_2_ atmosphere incubator at 37°C, the cells were examined using an inverted microscope equipped with a CCD camera. A colony is defined as a cluster of at least 60 cells, and visible colonies were counted. Cells were then washed with PBS twice, and counted using a haemocytometer.

### 2.4 Cell cycle and apoptosis assay

For the flow‐cytometric analysis of the cell cycle, cells were stained with propidium iodide (PI) using the Cycletest™ plus DNA reagent kit (BD Biosciences, Franklin lakes, NJ) according to the manufacturer's instructions. Data acquisition and analysis were performed using CellQuest and Modfit software on a flow cytometry (FACSCalibur, BD Biosciences, Mountain View, CA), respectively.

Apoptosis was determined using the Annexin V‐FITC/PI Detection kit (Beyotime Institute of Biotechnology, Haimen, Jiangsu, China) on a flow cytometry (Navios, Beckman Coulter, Brea, CA) according to the manufacturer's instruction.

### 2.5 Electron microscopy assay

After treatment with 20 μmol/L tigecycline for 48 hours, RPMI‐8226 cells were fixed in PBS (pH 7.4) containing 2.5% glutaraldehyde at 4°C for more than 2 hours. The cells were postfixed in OsO4 at room temperature for 60 minutes and were subsequently stained with 1% uranyl acetate, dehydrated through graded acetone solutions and embedded. Finally, the autophagosomes were observed under a transmission electron microscope (H‐7500, Hitachi, Japan).

### 2.6 Western blot analysis

After treatment with different concentrations of tigecycline with or without bafilomycin A1 (baf A1), the cells were collected and lysed immediately using RIPA lysis buffer (Beyotime Institute of Biotechnology) supplemented with PMSF and Halt protease and phosphatase inhibitor cocktail (Pierce, Rockford, IL). The protein was boiled for 8 minutes in 1× loading buffer and subjected to Western blot analysis using antibodies against SQSTM1/p62, LC3, p‐mTOR, mTOR, p‐AMPKa, AMPKa, p‐p70S6K, p70S6K, p‐4E‐BP1, 4E‐BP1 or GAPDH as reported previously.^19^ The bands were visualized by an enhanced chemiluminescence reagent (Thermo Fisher, Fremont, CA), and the optical densities of the bands were analysed using Image J software (NIH, Bethesda, MD).

### 2.7 In vivo studies

NOD/SCID mice (3‐4 weeks old/18‐20 g body weight) were purchased from Charles River (Charles River Laboratory Animal, Beijing, China). Exponentially growing RPMI‐8226 cells (5 × 10^6^) in 200 μL PBS were injected subcutaneously into the right flank of each mouse, which had been already intraperitoneally injected with cyclophosphamide (200 mg/kg) 24 hours before transplantation. When tumour volume reached approximately 300 mm^3^, the mice were randomized into four groups. The vehicle group was given saline, and the treatment groups were injected with tigecycline twice daily (75 mg/kg by intraperitoneal injection) or chloroquine daily (50 mg/kg by intraperitoneal injection) (Sigma‐Aldrich) or both drugs, respectively. Tumour length and width were measured every 2 days, and the volume was calculated using the formula: volume = length × width^2^ × 0.5236. All mice were sacrificed after 14 days. Animal procedures were carried out in accordance with institutional guidelines after Whenzhou Medical University Animal Care and Use Committee approved the study protocol.

### 2.8 Statistical analysis

The data are presented as mean ± SEM and analysed by one‐way ANOVA followed by post hoc Turkey's test to determine the differences between the groups. Differences were considered significant at *P *<* *0.05.

## 3 RESULTS

### 3.1 Tigecycline inhibits the proliferation and colony formation in MM cells

**Figure 1:**
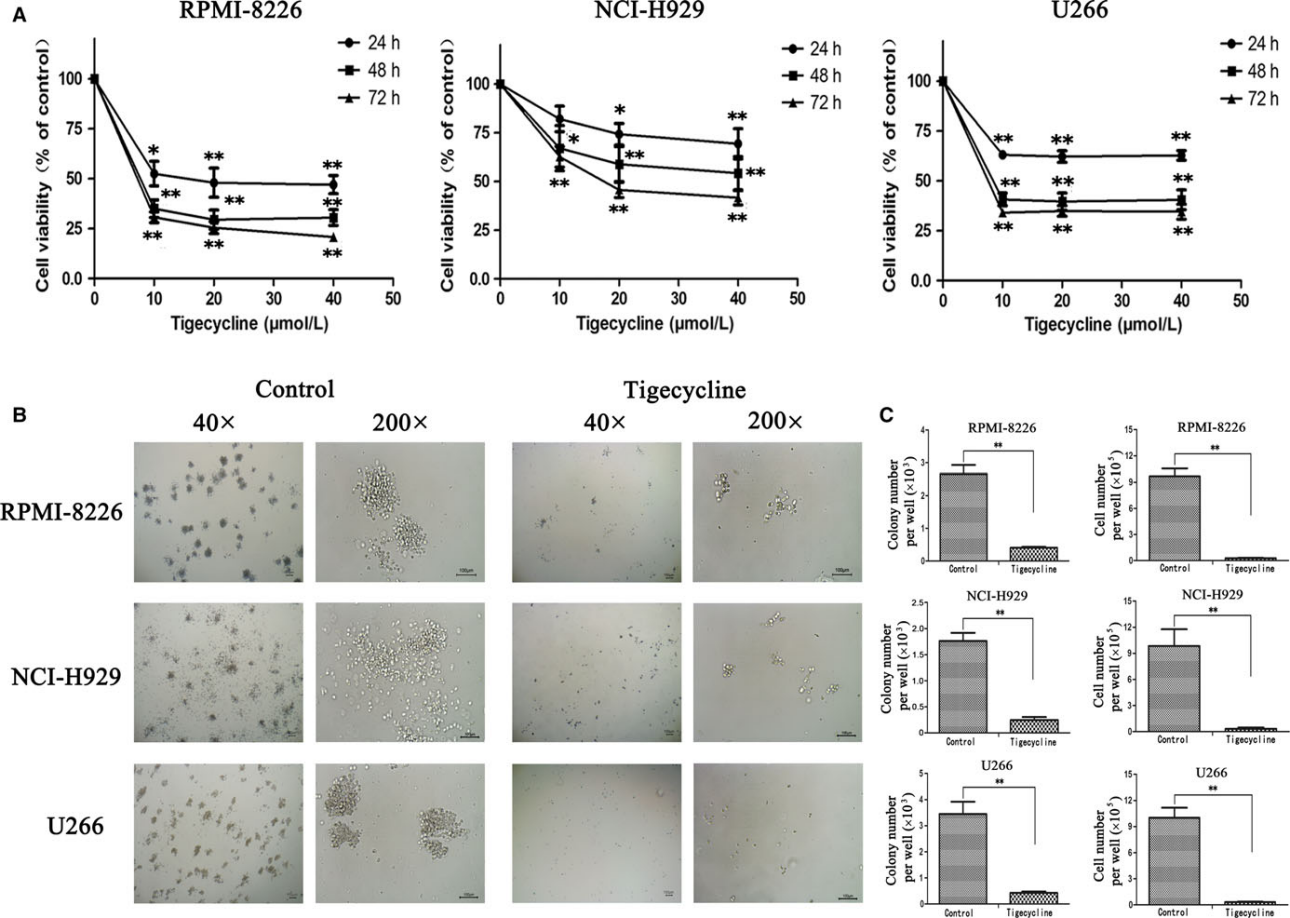
Tigecycline induces growth inhibition in MM cells. A, After cultured with different concentrations of tigecycline for 24, 48 and 72 h, the cell viability of RPMI‐8226, NCI‐H929 and U266 cells was measured by CCK‐8 assay. B, The morphological changes of colonies were observed under a microscopy in MM cell lines RPMI‐8226, NCI‐H929 and U266 treated with or without 20 μmol/L tigecycline for 7 d. Representative images were shown. C, Quantitative data of cell and colony number were presented in bar charts. Results were expressed as mean ± SEM representing at least three independent experiments. *P <* *0.05, **P <* *0.01 vs the respective control

Tigecycline has been reported to possess a potent antitumour activity against multiple solid or haematological malignancies, which arouses our interest to investigate whether tigecycline has a similar antitumour effect on MM. As expected, tigecycline dramatically impaired the cell viabilities of three MM cell lines (RPMI‐8226, NCI‐H929, and U266) tested in a time‐ and dose‐dependent manner (Figure 1A). The soft agar clone formation assay is a technique widely used to assess the survival and tumorigenic capabilities of tumour cells.^20^ The parallel effects were observed in soft agar assays with the above three cell lines. Obviously, tigecycline at 20 μmol/L considerably inhibited the colony formation, characterized by small colony size, compared with the vehicle cells (Figure 1B). Both colony number and total cell number were significantly reduced by tigecycline in all three MM cell lines tested (Figure 1C). These data suggested that tigecycline potently inhibits the proliferation and colony formation in MM cells.

### 3.2 Tigecycline induces cell cycle arrest at G0/G1 phase in MM cells

**Figure 2:**
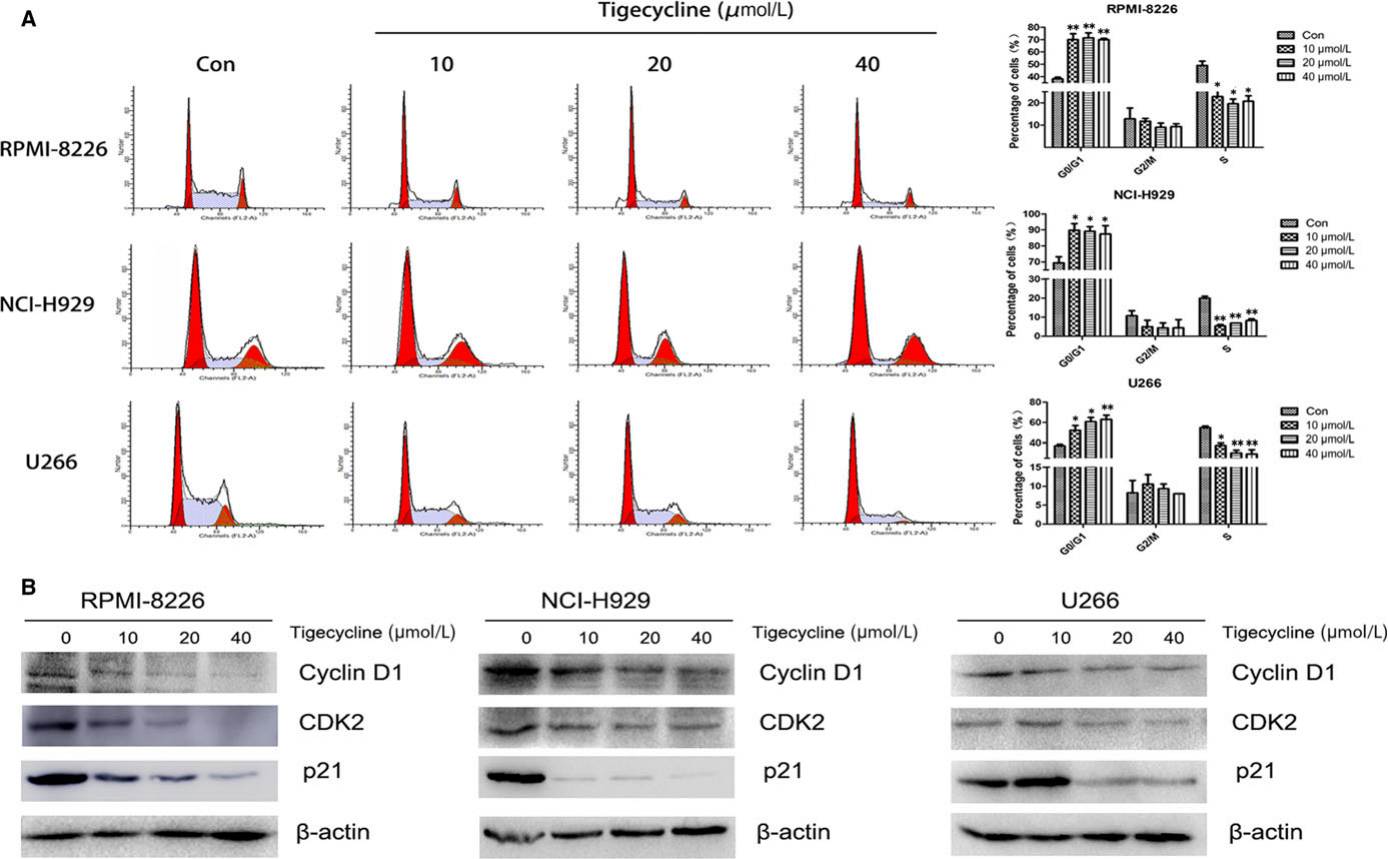
Tigecycline induces cell cycle arrest in MM cells. A, The cell cycle was analysed using PI staining by flow cytometry in MM cells treated with various concentrations (0, 10, 20, 40 μmol/L) of tigecycline for 48 h, and the percentages of cells in different phases were presented in bar charts. B, MM cells were treated with tigecycline as above, and Western blot analysis was performed to assess the level of p21, cyclin D1 and CDK2. *P <* *0.05, **P <* *0.01, vs the respective control

As it has been reported that tigecycline impairs the cell viability mainly through inducing cell cycle arrest rather than apoptosis,^11^ we first analysed the effect of tigecycline on the cell cycle of MM cells and found that tigecycline treatment led to an increase in G0/G1 phase with diminished S phase (Figure 2A), suggesting that tigecycline is capable of inducing G0/G1 arrest to decelerate the cell cycle, and preventing the cells from entering the S phase and proliferating. As CDK2 is one of the key kinases controlling G1/S transition and DNA replication and p21 is a critical regulator of CDK2, we measured these two proteins and found that tigecycline markedly decreased the levels of CDK2 and p21 in three MM cell lines RPMI‐8226, NCI‐H929, and U266 (Figure 2B). Cyclin D1, a major cyclin driving the G1/S phase transition,^21^ was also dramatically decreased by tigecycline in all three MM cell lines tested (Figure 2B). We next analysed whether tigecycline induces apoptosis of MM cells and found that tigecycline almost did not induce apoptosis in RPMI‐8226 cells (Figure S1). These results strongly indicated that tigecycline impairs the cell viability of MM cells mainly because of cell cycle arrest at G0/G1 phase rather than apoptosis induction.

### 3.3 Tigecycline induces cytoprotective autophagy in MM cells

**Figure 3:**
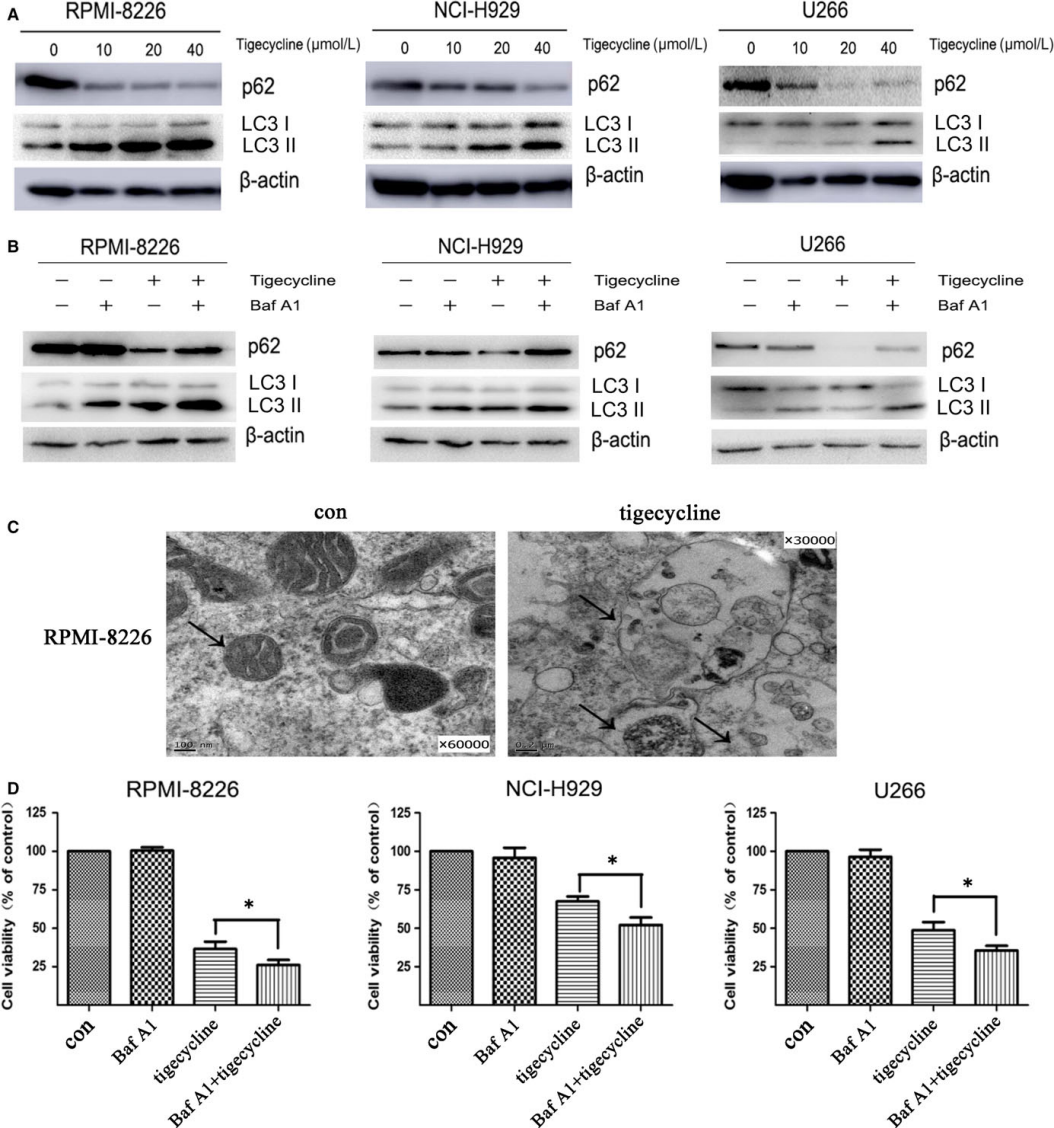
Tigecycline induces cytoprotective autophagy in MM cells. A, RPMI‐8226, NCI‐H929 and U266 cells were treated with 0, 10, 20, 40 μmol/L tigecycline for 48 h, the autophagy markers LC3 and SQSTM1/p62 were detected by Western blot analysis. B, MM cells were treated with 20 μmol/L tigecycline in the presence or absence of 10 nmol/L bafilomycin A1 (Baf A1) for 48 h, and then, the levels of LC3 and SQSTM1/p62 were determined by Western blot analysis. C, After treatment with 20 μmol/L tigecycline for 48 h, autophagosomes were observed in RPMI‐8226 cells under a transmission electron microscope. On the left panel, the arrow indicates the mitochondrial; on the right panel, the arrows indicate the autophagosomes. D, After treatment with 20 μmol/L tigecycline in the presence or absence of 10 nmol/L Baf A1 for 48 h, the cell viability of MM cells was measured by CCK‐8 assay. Results were expressed as mean ± SEM representing at least three independent experiments. *P <* *0.05, vs tigecycline alone group

Tigecycline has been demonstrated to induce autophagy in gastric cancer cells.^7^ To explore whether autophagy is also functionally involved in tigecycline‐induced cytotoxicity, we analysed the expression levels of LC3 and SQSTM1/p62 and found that tigecycline dose‐dependently promoted the accumulation of LC3 II and subsequently degraded the autophagy substrate SQSTM1/p62 in MM cell lines RPMI‐8226, NCI‐H929 and U266 (Figure 3A), indicating that autophagy does occur in tigecycline‐treated MM cells. To further confirm whether tigecycline induces autophagy in MM cells, an autophagy inhibitor Baf A1, the lysosomotropic agent that inhibits lysosomal degradation of autophagosome was employed. Inhibition of autophagy at an early stage results in decreased production of LC3 II, but inhibition of autophagic flux at a late stage using Baf A1 or chloroquine leads to increased levels of LC3 II.^22^ As expected, when combined with Baf A1, the attenuation of SQSTM1/p62 was partly reversed with the level of LC3 II still gradually increasing, revealing that Baf A1 delayed the tigecycline‐induced autophagy (Figure 3B). Concurrently, autophagosomes were also observed in RPMI‐8226 cells treated by tigecycline (Figure 3C). Thus, autophagy indeed occurred in tigecycline‐treated MM cells. Subsequently, we investigated the role of autophagy in anti‐MM effects of tigecycline. Tigecycline‐induced impairment of cell viability was aggravated by treatment with Baf A1 in all three MM cells tested (Figure 3D), suggesting that autophagy plays a cytoprotective role in tigecycline‐induced cytotoxicity. These findings strongly suggested that a cytoprotective autophagy does occur in MM cells treated by tigecycline.

### 3.4 AMPK/mTOR signalling pathway is involved in tigecycline‐induced autophagy in MM cells

**Figure 4:**
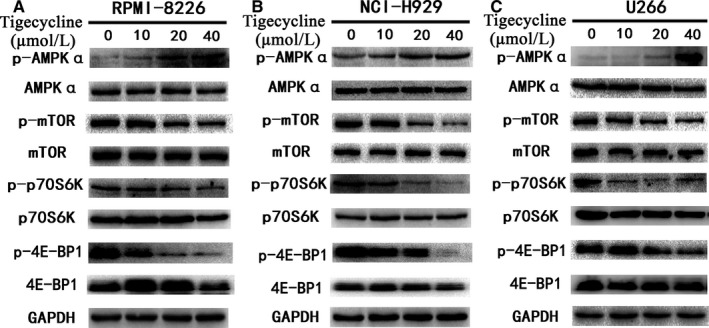
AMPK/mTOR pathway is implicated in tigecycline‐induced autophagy in MM cells. After treated with various concentrations of tigecycline for 48 h, the levels of AMPKa, mTOR, p70S6K, and 4E‐BP1, as well as the phosphorylation of these proteins were analysed by Western blot in MM cell lines RPMI‐8226 (A), NCI‐H929 (B) and U266 (C). Images shown were representatives of at least three independent experiments

The mTOR signalling pathway serves as a central regulator of cell metabolism, growth, proliferation and survival. Recent studies have also revealed that mTOR signalling is tightly related to autophagy.^23^ The mTOR kinase downstream targets the eukaryotic initiation factor 4E‐BP1 and the p70S6K1. Inhibition of mTOR signalling leads to dephosphorylation of 4E‐BP1 and p70S6K1 to induce autophagy. Therefore, we firstly evaluated the phosphorylation of these three proteins and found that tigecycline dramatically inhibited the phosphorylation of mTOR and two downstream effectors 4E‐BP1 and p70S6K in three MM cell lines RPMI‐8226 (Figure 4A), NCI‐H929 (Figure 4B) and U266 (Figure 4C). As the upstream positive regulatory role of Akt in mTOR activation has been reported,^19^ we evaluated the Akt phosphorylation and found that tigecycline slightly elevated the Akt phosphorylation in NCI‐H929 cells (Figure S2), suggesting that tigecycline‐induce mTOR signalling inhibition is not through directly influencing the Akt signalling. We subsequently analysed the phosphorylation of AMPK, another important signalling pathway can regulate the mTOR signalling,^24^, ^25^ and found that tigecycline significantly enhanced the phosphorylation of AMPK in all three MM cell lines tested (Figure 4). These findings manifested that tigecycline promoted autophagy in MM cells mainly through regulating the AMPK/mTOR signalling but not Akt signalling.

### 3.5 Autophagy inhibitor chloroquine synergistically enhances anti‐MM efficacy of tigecycline in vivo

**Figure 5:**
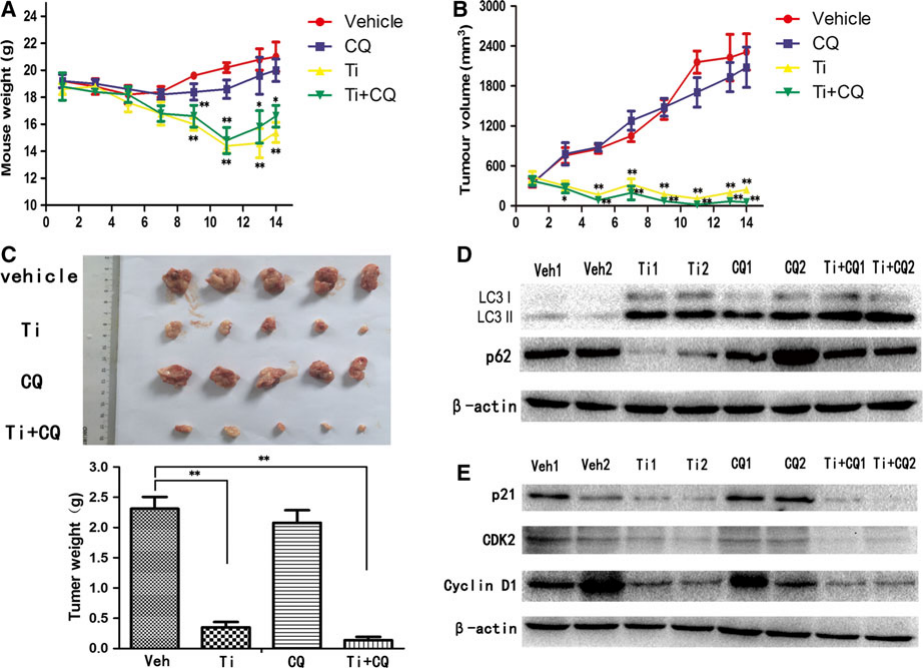
Autophagy inhibition enhances the antitumour efficacy of tigecycline in a MM xenograft. (A,B) RPMI‐8226 cells (5 × 10^6^) were injected subcutaneously into the right flank of female NOD/SCID mice (n = 5 for each group). When the tumours reached approximately 300 mm^3^ in volume, intraperitoneal injections of tigecycline (Ti; 75 mg/kg, twice a day) or chloroquine (CQ; 50 mg/kg, daily), or saline (Veh), or both Ti and CQ were administered for 14 d, tumour volume and the body weight were measured every other day. C, Size and weight of xenograft tumour were elevated after mice were killed. D, The levels of LC3 and SQSTM1/p62 in tumour tissues were determined by Western blot. E, The levels of p21, cyclin D1 and CDK2 in tumour tissues were determined using Western blot analysis. Results were expressed as mean ± SEM, and images shown were representatives of at least three independent experiments. *P <* *0.05, **P <* *0.01, vs tigecycline alone group

To investigate whether tigecycline also potently inhibits MM cells in vivo, we established a xenograft model in NOD/SCID mice by subcutaneous injection of RPMI‐8226 cells. When tumour volume reached approximately 300 mm^3^, the mice were randomized into four groups. One group was treated with vehicle, while others were administrated with tigecycline or chloroquine or both drugs. Chloroquine, similarly to Baf A1, is an autophagy inhibitor blocking autophagy flux at late stage. Only a moderate reduction on body weight in tigecycline‐treated groups was observed (Figure 5A). And tigecycline delayed tumour growth beginning at 2 days after the initial treatment (Figure 5B). After 2 weeks of daily treatment with tigecycline, tumour size and weight were obviously reduced compared to the vehicle. Furthermore, the combination treatment of tigecycline and chloroquine demonstrated a noticeable (although not significant) decrease in subcutaneous tumours compared with mice that received tigecycline alone (Figure 5B,C), suggesting that autophagy plays a protective role in anti‐MM effects of tigecycline. Similarly to in vitro experiments, tigecycline elevated the LC3 II level, accompanied by the down‐regulation of SQSTM1/p62 in tumour tissue in tigecycline‐treated group (Figure 5D), indicating that autophagy does occur in tigecycline‐treated MM cells in vivo. Concurrently, the levels of G1/S transition‐associated proteins p21, cyclin D1 and CDK2 were also significantly decreased in tumour tissue compared with mice that received vehicle (Figure 5E). These results suggested that tigecycline exhibits a potent anti‐MM effect in tumour xenograft mice through inducing cell cycle arrest at G0/G1 phase, and autophagy inhibitor chloroquine has a synergetic effect with tigecycline treatment.

## 4 DISCUSSION

The prognosis of patients with MM is still dismal despite improved remissions with novel agents. Tigecycline as a FDA‐approved antibiotic can inhibit the synthesis of bacterial proteins and mitochondrial protein translation. Recently, it has been reported that tigecycline alone, or in combination with other therapeutic agents, can effectively kill, even eradicate multiple solid tumours and haematological malignancies.^26^, ^27^ In the present study, we demonstrated that tigecycline potently impaired the proliferation in MM cells in a dose‐dependent manner. Furthermore, the soft agar assay displayed that tigecycline dampened survival and self‐renewal of MM cells in vitro. Tumour xenograft experiment in NOD/SCID mice indicated that tigecycline dramatically attenuated MM tumour growth in vivo. Flow cytometry demonstrated that tigecycline induced cell cycle arrest at G0/G1 phase rather than apoptosis. Western blot analysis with decreased expression of CDK2, p21 and cyclin D1 further verified that tigecycline induced cell cycle arrest at G1 phase in MM cells. Our results intensively indicated that tigecycline inhibited cell proliferation and growth via inducing cell cycle arrest at G0/G1 phase in MM cells.

In our present study, tigecycline treatment led to the accumulation of LC3 II and the degradation of SQSTM1/p62, with increased autophagosomes observed under transmission electron microscope, suggesting that autophagy does occur in tigecycline‐treated MM cells. Autophagy is an evolutionarily conserved self‐digestive recycling pathway whose prime function is to maintain cell metabolism under nutrient deprivation. During plasma cell differentiation, selective autophagy of endoplasmic reticulum restricts the expression of the transcriptional repressor Blimp‐1 and the secretion of immunolglobulins (Ig), thereby optimizing energy metabolism and extending plasma cell lifespan.^28^ Therefore, autophagy as a physiological modulator can ensure sustainable Ig production by regulating unfolded protein response‐driven expansion of the secretory apparatus in plasma cells.^29^ To ascertain the role of tigecycline‐induced autophagy in MM cells, we used Baf A1 as an in vitro autophagy inhibitor and chloroquine as an in vivo autophagy inhibitor. These two results revealed that autophagy acted as a cytoprotective role in tigecycline‐treated MM cells. Combination of autophagy inhibitor with tigecycline has a stronger antitumour effect on MM cells than tigecycline alone.

The molecular mechanism of autophagy involves several conserved Atg proteins. In the present study, we only simply detected the level of LC3 II and its degradation substrate SQSTM1/p62 to confirm the occurrence of autophagy. We mainly clarified the upstream mechanisms of autophagy induction and found that the activation of AMPK is implicated in tigecycline‐induced autophagy. In contrast to normal cells, MM cells relied heavily on glycolysis and mitochondrial respiration to meet the high demands of energy production and metabolism.^30^, ^31^, ^32^ Tigecycline as a mitochondrial protein translation inhibitor leads to energy deprivation, and AMPK is a crucial cellular energy sensor. The activation of AMPK subsequently resulted in the inhibition of mTOR, which can regulate protein synthesis, cell growth and proliferation through its downstream effectors 4E‐BP1 and p70S6K1. Targeting mTOR has been proved to be effective for MM treatment.^33^ Concurrently, increased activation of Akt may be because of a marked decrease in mTOR activity induced by tigecycline in MM cells.

In conclusion, we first showed that tigecycline inhibited MM cells proliferation and growth both in vitro and in vivo through inducing cell cycle arrest at G0/G1 phase. Second, we found that autophagy did occur and exerted a cytoprotective role in tigecycline‐treated MM cells, and autophagy inhibitor could enhance the efficacy of tigecycline. Therefore, it is highlighted that combination of autophagy inhibitor and tigecycline might be a promising therapeutic strategy for MM.

## CONFLICT OF INTEREST STATEMENT

The authors have no conflict of interest.

## Supporting information


** **

** **
