# Supplementary figures and images for: Inhibition of autophagy enhances the antitumour activity of tigecycline in multiple myeloma
Source: J Cell Mol Med. 2018 Sep 24;22(12):5955–63. doi: 10.1111/jcmm.13865 (PMC6237591; doi:10.1111/jcmm.13865)

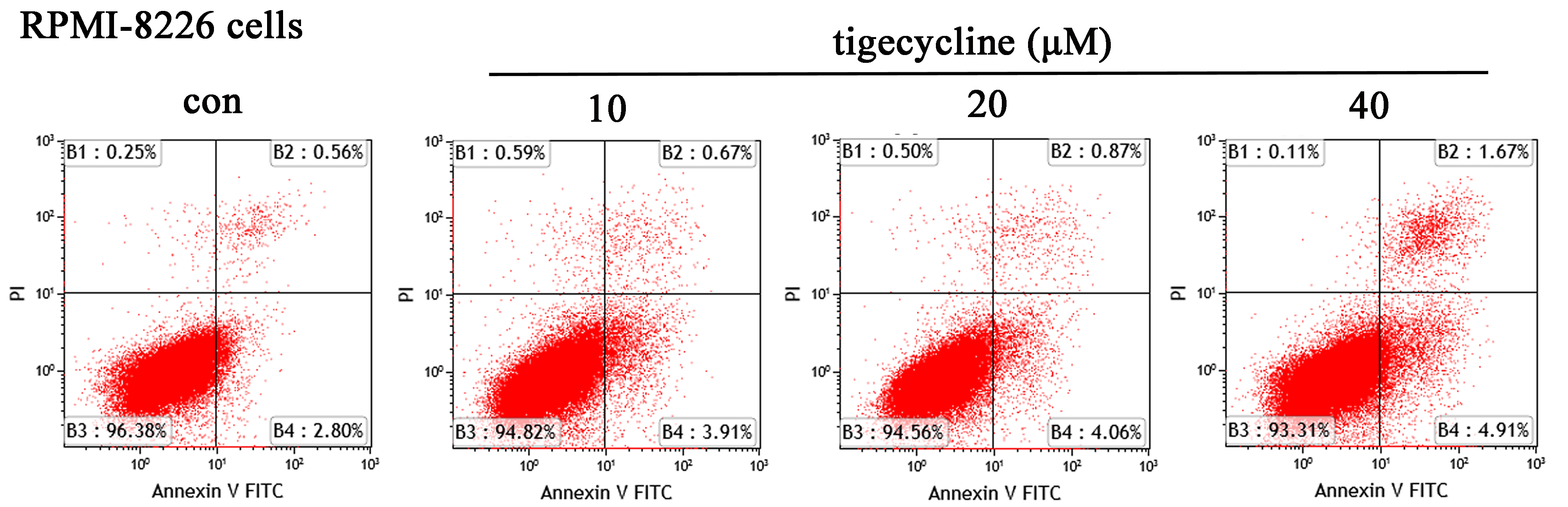

Supplement: Supplementary file 1 [file JCMM-22-5955-s001.tif]

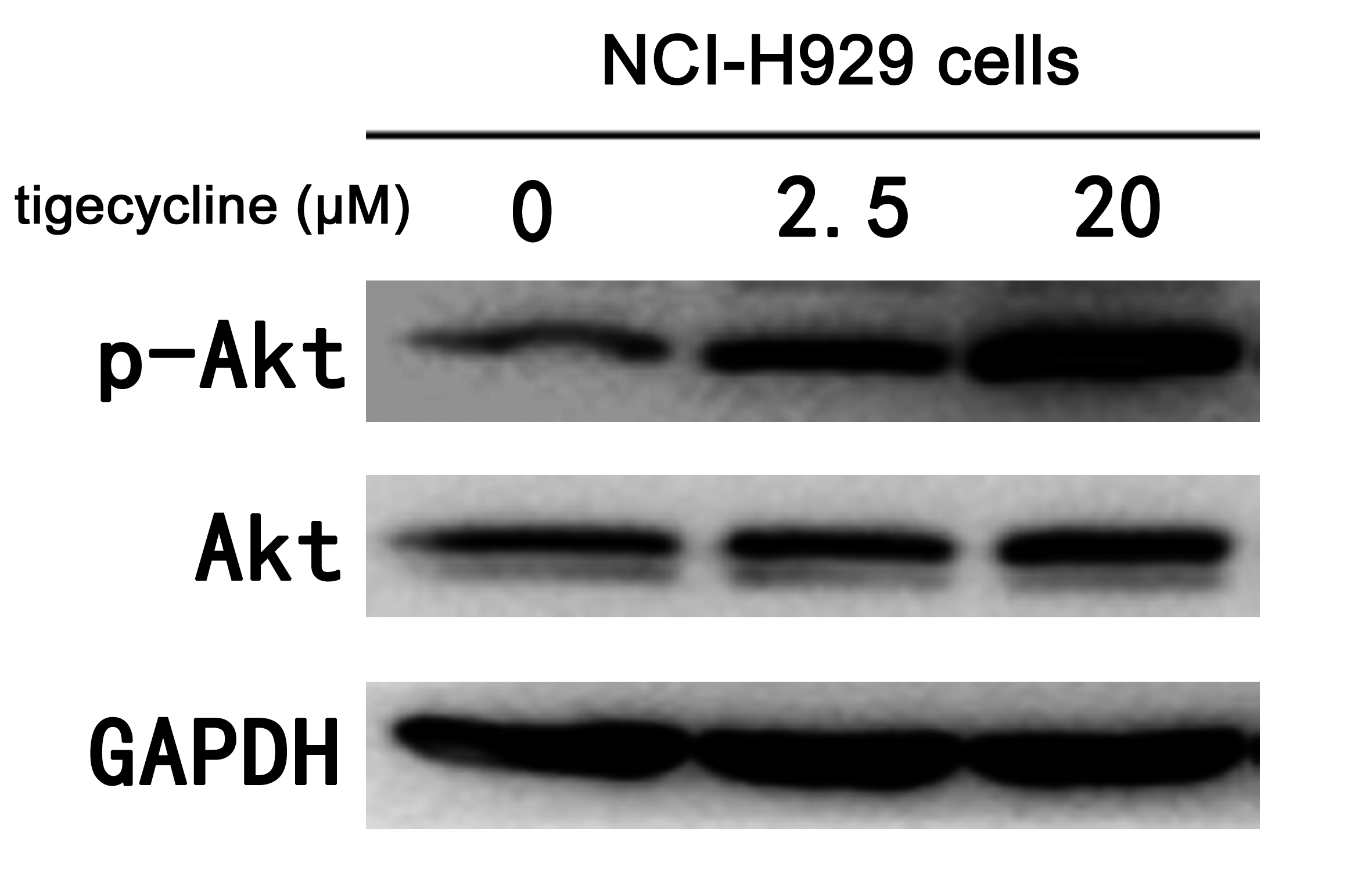

Supplement: Supplementary file 2 [file JCMM-22-5955-s002.tif]
